# Inhibition of Caveolin-1 Restores Myeloid Cell Function in Human Glioblastoma

**DOI:** 10.1371/journal.pone.0077397

**Published:** 2013-10-09

**Authors:** Shinji Shimato, Lisa M. Anderson, Martin Asslaber, Jeffrey N. Bruce, Peter Canoll, David E. Anderson, Richard C. E. Anderson

**Affiliations:** 1 Department of Neurosurgery, Gabriele Bartoli Brain Tumor Research Laboratory, Columbia University, New York, New York, United States of America; 2 Erinyes Biotechnologies, LLC, Boston, Massachusetts, United States of America; 3 Department of Pathology, Medical University of Graz, Graz, Austria; 4 Department of Pathology, Columbia University, New York, New York, United States of America; The Ohio State University Medical Center, United States of America

## Abstract

**Background:**

Gliomas are the most common primary brain tumor in both children and adults. The prognosis for glioblastoma (GBM), the most common type of malignant glioma, has remained dismal, with median survival a little over one year despite maximal therapy with surgery, chemotherapy, and radiation. Although immunotherapy has become increasingly successful against many systemic tumors, clinical efficacy against brain tumors has been limited. One reason for this is an incomplete understanding of the local immunologic tumor microenvironment, particularly the function of large numbers of infiltrating myeloid derived cells. Monocytes/microglia are myeloid derived immunomodulatory cells, and they represent the predominant infiltrating immune cell population in gliomas. Our group has previously demonstrated using complementary *in* *vitro* and *in* *vivo* approaches that GBM tumor cells polarize tumor-associated myeloid cells (TAMs) and suppress their immunostimulatory function.

**Methods and Results:**

To better understand the mechanisms responsible for this immunosuppression, we used gene expression profiling of stimulated monocytes in the presence or absence of GBM tumor cells. Our analysis identified caveolin-1 (CAV1), a plasma membrane molecule with pleiotropic functions, as significantly up-regulated in monocytes in the presence of GBMs. We validated these findings *ex* *vivo* by confirming up-regulation of CAV1 in TAMs isolated from GBMs immediately after surgical resection. Finally, we demonstrate that siRNA inhibition of CAV1 restores myeloid cell function, as measured by TNF-alpha secretion, in the presence of GBMs.

**Conclusions:**

Restoration of TAM function through pharmacologic blockage of CAV1 may facilitate more successful immunotherapeutic strategies directed against a variety of solid human tumors infiltrated by TAMs.

## Introduction

Currently, the majority of research involving the immunology of malignant gliomas focuses on T cells. This is in part because there is clear evidence that T cells infiltrate glioblastomas (GBMs) [1-5] and also because of increasing success with dendritic cell-based vaccinations to boost cytotoxic T cell responses (reviewed in 6). However, there is increasing awareness that myeloid derived immune cells play a significant role in the local tumor microenvironment. In both animal models and cancer patients, for example, myeloid-derived suppressor cells have been implicated in the generation and propagation of local tumor immunosuppression [7]. In the central nervous system, until recently [8,9] the contribution of myeloid cells in the regulation of the local immune response to GBMs has largely been overlooked. 

Our group and others have reported that tumor associated myeloid cells (TAMs) greatly outnumber any other immune cell type in human gliomas including T cells [8-13]. We recently demonstrated that GBM tumor cells inhibit the ability of myeloid cells to respond to a wide array of potent stimuli *in vitro*, and moreover, can render them tolerogenic [14]. To determine if our observations regarding the frequency and functional impairment of myeloid cells in human GBMs were also present *in vivo*, we developed a novel animal model by injecting PDGF-driven murine glioma cells into the white matter of mice [15]. Similar to both our findings *in vitro* and from human GBMs *ex vivo*, TAMs from our murine model were found at high frequency but with significant functional impairment [11]. 

To determine if a single, dominant molecule was responsible for the GBM-mediated suppression of TAM function, we performed a comprehensive, array-based approach to identify pathways and mechanisms by which GBMs suppress myeloid cell activation. Our results identified caveolin-1 (CAV1) as significantly up-regulated in myeloid cells in the presence of GBM tumor cells. We then validated this finding *ex vivo* by confirming upregulation of CAV1 in TAMs isolated from GBMs immediately after surgical resection. Finally, we demonstrated that blocking CAV1 with siRNA inhibition restored myeloid cell function, as measured by TNF-alpha secretion, in the presence of GBMs.

## Materials and Methods

### Monocyte and PBMC extraction

Peripheral blood mononuclear cells (PBMC) were isolated from freshly isolated blood from healthy subjects. Under approval from the Columbia University Institutional Review Board (IRB) (protocol IRB-AAAA4666), blood samples and brain tumor specimens are collected from patients by a broker and deposited in an institutional tumor bank. Specimens are provided to Columbia University personnel upon request after complete de-identification. As per the above protocol, further research with these de-identified specimens is classified as NOT human-related research and no further IRB approval is required. Healthy subjects were used to obtain normal peripheral blood monocytes for use in these studies. The first group was used for the microarray study and consisted of both men (n = 4) and women (n = 4) with a mean age of 44 years. A separate group of healthy subjects was used to confirm the microarray data and consisted of both men (n = 3) and women (n = 2) with a mean age of 41 years. Blood was drawn into vacutainers containing lithium-heparin anti-coagulant (BD Biosciences). PBMCs were extracted using Ficoll density gradient centrifugation. Monocytes were then isolated from PBMCs by negative selection using immunomagnetic beads (Miltenyi Biotec, Auburn, CA). 

### Co-culture assays and re-sorting of monocytes following co-culture

*Ex vivo* monocytes (2x10^6^ cells/well) were cultured alone or with a primary GBM tumor cell line (RCA; obtained by culture and expansion of an *ex vivo* tumor specimen in cell culture medium [14]) at a ratio of 2:1 monocytes:GBM in polystyrene 2.5 ml tubes in the absence or presence of lipopolysaccharide (LPS; 1ug/ml). After 4 hours of co-culturing in endotoxin free conditions, monocytes were stained with CD11b-PE monoclonal mouse anti-human antibody (BD Pharmingen, San Diego, CA) and a FACSAria (Becton Dickinson, San Diego, CA) cell sorter was used to isolate monocytes (CD11b+) to obtain a highly purified cell population (>99% purity). Adult normal human astrocytes were purchased from two commercial sources (Cambrex/Lonza; All Cells) and cultured in Astrocytes Basal Media supplemented with astrocyte growth medium SingleQuots (Lonza) [14]. 

### Gene expression analysis

Applied Biosystems Human Genome Survey Arrays V2.0 were used to determine the transcriptional profiles of 26 samples. DIG-UTP labeled cRNA was generated and linearly amplified from 500 ng total RNA using the Chemiluminescent RT-IVT Labeling Kit v 2.0 (Applied Biosystems, Foster City, CA, US) as described by the protocol. Array hybridization, chemiluminescence detection, image acquisition and analysis were performed using Applied Biosystems Chemiluminescence Detection Kit and Applied Biosystems 1700 Chemiluminescence Microarray Analyzer following the manufacturer’s instructions. Each microarray was pre-hybridized at 55°C for 1hr in hybridization buffer with blocking reagent. Between 4 and 20 ug DIG-labeled cRNA targets were first fragmented, mixed with internal control target and hybridized to the prehybridized microarrays in a volume of 1.5ml at 55°C for 16 hrs. After hybridization, the arrays were washed with hybridization wash buffer and chemiluminescence rinse buffer. Enhanced chemiluminescent signals were generated by incubating arrays with Alkaline Phosphatase conjugated anti-digoxigenin antibody followed by incubation with Chemiluminescence Enhancing Solution and a final addition of Chemiluminescence Substrate. Four images were collected for each microarray using the ABI 1700 Chemiluminescent Microarray Analyzer. Images were auto-gridded and the chemiluminescent signals were quantified, corrected for background and spot and spatially normalized.

### Statistical analysis

The entire statistical analysis was performed within "R" software environment (www.r-project.org). The ABarray package (Yongming Andrew Sun, Applied Biosystems) from Bioconductor (www.bioconductor.org) was used for quality control purposes and quantile normalization. 14414 genes showed reliable expression values throughout all comparisons based on standard filter criteria of the ABarray package. One sample had to be omitted from further analysis because of low number of probes detectable and low detection concordance. The Pairwise Local Pooled Error Test (PLPE package from Bioconductor) was performed in order to compare gene expression values of unstimulated monocytes against monocytes stimulated with LPS (1), stimulated monocytes against stimulated monocytes cocultured with glioblastoma cells (2) and stimulated monocytes cocultured with glioblastoma cells against stimulated monocytes cocultured with human astrocytes (3). Genes were filtered by FDR < 0.001 of the Paired L-statistic or Paired Lw-statistic for the second comparison and FDR < 0.01 of the third comparison. The majority of these genes were not affected by LPS stimulation (FDR > 0.01 in first comparison).

### Collection of human tumors and processing of ex vivo specimens

Fresh human tumor and normal brain specimens were obtained according to institutional review board guidelines (see above). Normal brain specimens were isolated from the anterior temporal tip in patients undergoing non-tumor temporal lobe epilepsy surgery. All tumor specimens were classified by neuropathogists as WHO grade IV glioblastomas. Nine patients had classical type GBM and one had GBM with oligodendroglial components. After specimens were collected in the operating room, they were transported and mechanically dissociated on ice in serum-free medium (modified Eagle’s medium containing 20nmol/L 4-(2-hydroxyethyl)-1-piper-azineethanesulfonic acid) and enzymatically digested using DNase (100ug/mL; Sigma Chemical Co., St. Louis, MO) and trypsin for 20 minutes. Single-cell suspensions were obtained by passing tumor slurries through a 100-um filter. Tumor cells were purified by density centrifugation using 30% sucrose solution for 20 minutes at 4500 rpm. The cells were washed in PBS twice and used for cell sorting.

### Cell sorting

Cell sorting was performed to obtain purified TAM populations from single-cell suspensions from *ex vivo* specimens. Cells were placed in PBS with 2% fetal bovine serum and allowed to incubate with anti-CD11c-PE antibody and anti-CD11b-APC antibody for 20 minutes at room temperature. Cells were washed, resuspended in PBM containing 2% fetal bovine serum with 1:1000 dilution of 4’,6-diamidino-2-phenylindole (DAPI; Dako, Fort Collins, CO). Cells were distinguished from debris and dead cells on the basis of appropriate scatter properties. Cells included in the range of DAPI^-^, CD11b^+^, and CD11c^+^ were collected as the TAM population [16] and used for mRNA analysis.

### siRNA transfection

siRNA transfection was performed to knock down each candidate gene in a monocyte cell line (THP-1 cells [Public Health England]). 24 hours before transfection, THP-1 cells were plated in 6-well plate at 5 x 10^5^ per well in RPMI1640 containing 10% FBS and PMA (10^-8^M) with no antibiotics. Transfection was done with Lipofectamine RNAiMAX (Invitrogen) with siRNA at a final concentration of 10nM according to the manufacturer’s recommendation. After 24hr-incubation, all cells were detached by trypsin and collected and used for coculture experiments. Some of the cells were used for extracting RNA for mRNA analysis of each candidate gene. All siRNAs were synthesized by Ambion (Darmstadt, Germany) and were purchased as annealed RNA-duplexes.

### mRNA expression analysis

mRNA expression analysis was done for *ex vivo* sorted TAM samples and THP-1 cells transfected with siRNA. Total RNA from the samples was obtained using an RNeasy Mini Kit (Qiagen) following the manufacturer’s instructions. Synthesis of cDNA was carried out using Oligo(dt) primers and Superscript-II reverse transcriptase according to the manufacturer’s specifications (Invitrogen). Real-time PCR was performed in duplicate reactions employing ABI PRISM 7300 (Applied Biosystems, Darmstadt, Germany) with standard conditions. RNA levels reported are relative to GAPDH, normalized using the equation 1/2^ΔCt^. All primers and probes were purchased from Applied Biosystems.

### Coculture assays

THP-1 (1 X 10^5^) cells transfected with siRNA were cultured alone or with 1 X 10^5^ tumor cells from a primary GBM cell line [14][17]) in duplicate in round-bottom 96-well plate in RPMI 1640 supplemented with 10% FBS. LPS was added at 1ug/ml as a stimulus. After 24-hr incubation, all supernatants were collected and used for ELISA for determining TNF concentration.

## Results and Discussion

### Gene expression profiling

**Figure 1:**
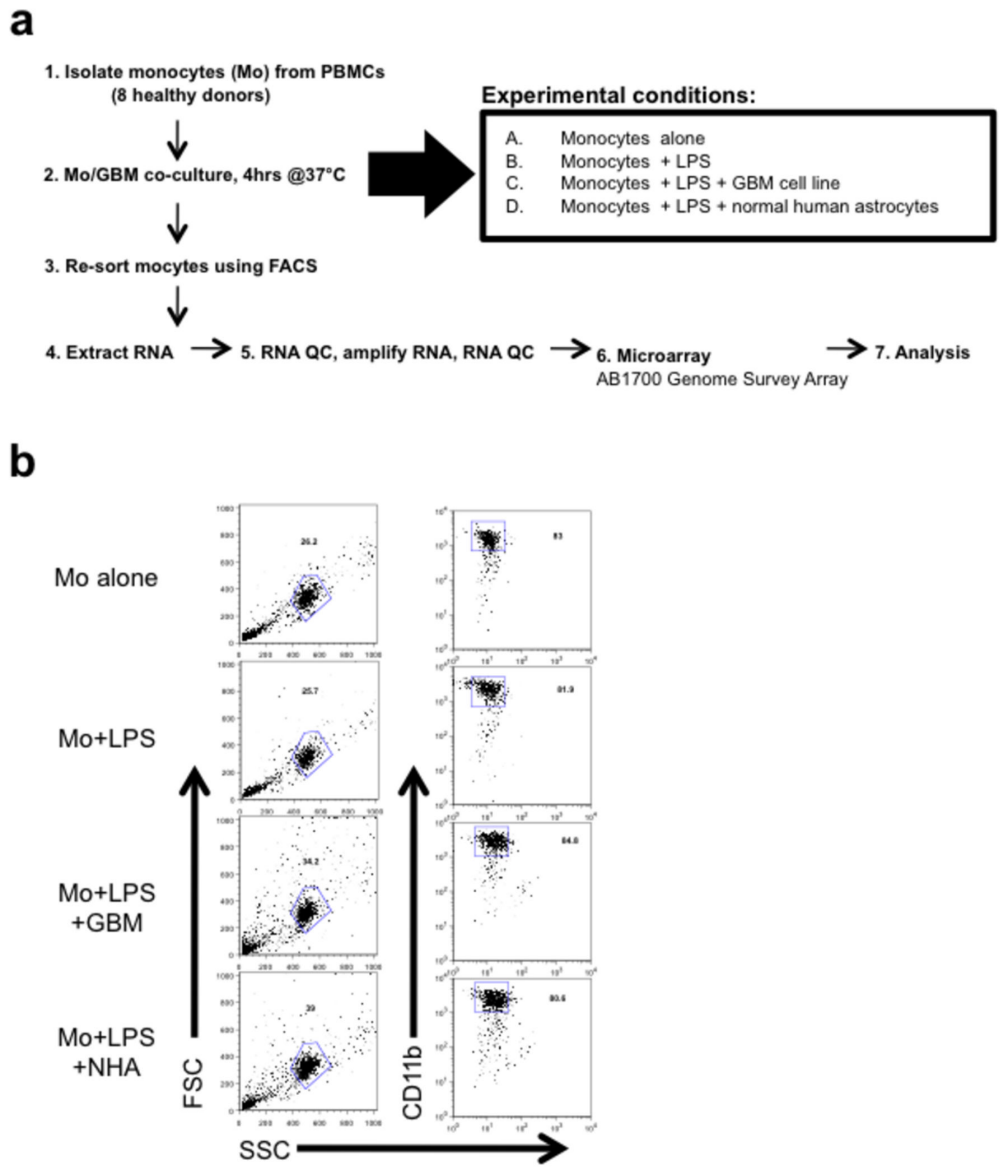
Experimental design and sample processing for microarray analysis of GBM-induced molecules in co-cultured myeloid cells. (**a**) ABI1700 cDNA microarrays were used to analyze global changes in gene transcripts using a cutoff in the change of gene expression of > 2 fold. We analyzed the expression profile of 29,362 human genes using RNA samples from unstimulated monocytes (n=8), LPS-stimulated monocytes (n=8), LPS-stimulated monocytes cocultured with glioblastoma cells (n=8), and monocytes cocultured with human astrocytes (n=2). Of these, 14,414 gene expression profiles were used for subsequent analysis, as this set of genes passed all quality control criteria. Local Pooled Error Tests (PLPE package from Bioconductor) were performed comparing gene expression values of monocytes stimulated with LPS in the absence vs. presence of GBM cells and subsequently stimulated monocytes co-cultured with GBM cells vs. stimulated monocytes co-cultured with primary human astrocytes. (**b**) After 4 hours of culture in 4 experimental conditions, monocytes were isolated from GBM or human astrocytes based on FSC/SSC properties. Purity was re-confirmed in a small fraction of each sample based on CD11b expression.

**Figure 2:**
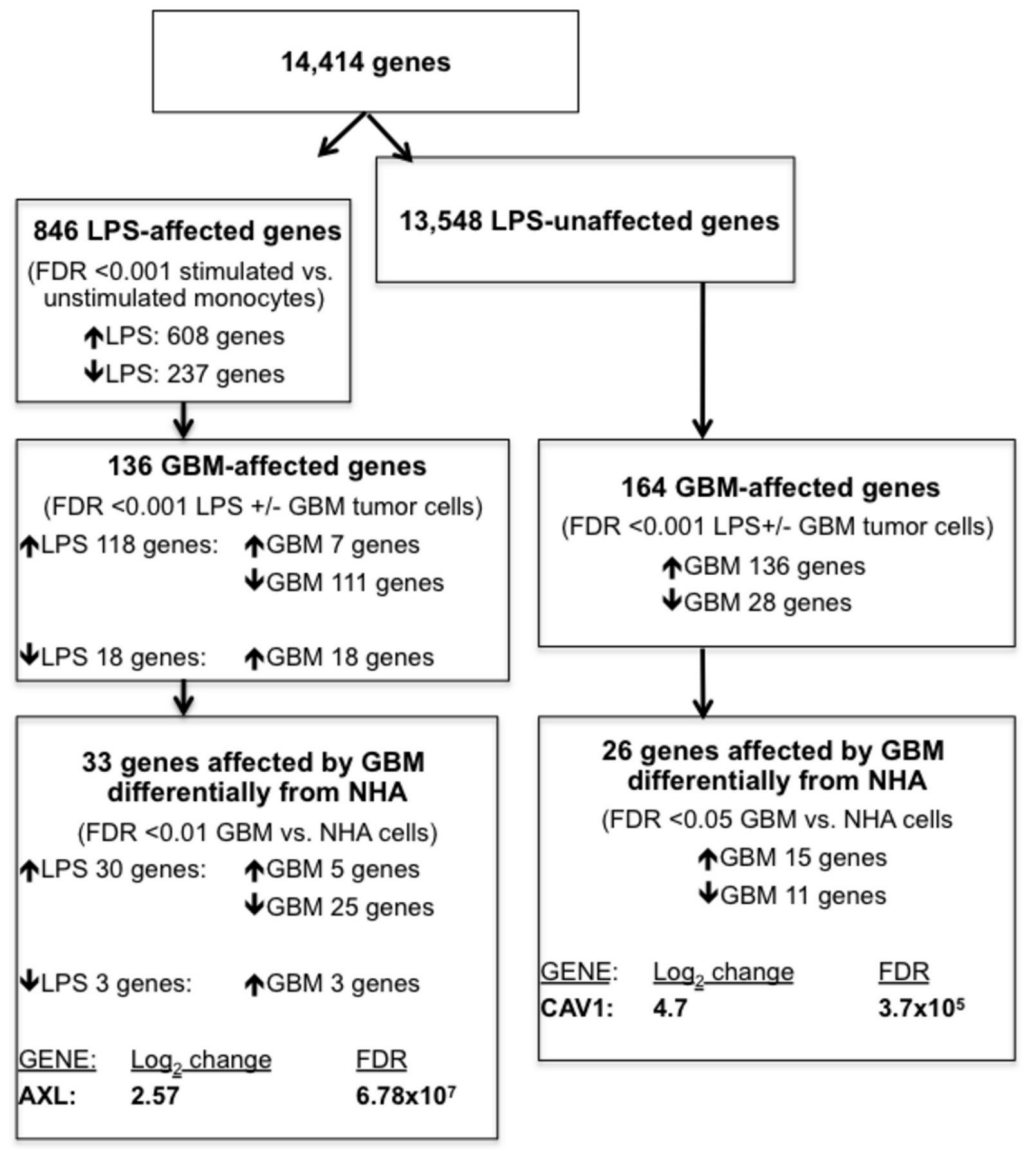
Flow schematic summarizing results of GBM-induced genes in co-cultured human myeloid cells. The number of genes satisfying each selection criteria are summarized, resulting in two gene subsets: those affected by LPS stimulation as well as culture with GBM tumor cells but not non-transformed human astrocytes, exemplified by AXL, and those unaffected by LPS stimulation but affected by GBM tumor cells, exemplified by CAV1. Additional genes within these two gene susbsets included CDCP1, CKS2, STC1, KRT18, and PHLDA2, but their differential expression could not be independently replicated (data not shown).

Human monocytes from eight healthy donors were freshly isolated and left unstimulated or stimulated with LPS under three different conditions: alone or in the presence of GBM tumor cells or normal human astrocytes (Figure **1a**). Unlike many other GBM microarray studies, we next re-isolated monocytes using Fluorescence-activated Cell Sorting (FACS) based on forward scatter (FSC) and side scatter (SSC) properties that were distinct from the GBM or astrocytes. We then confirmed purity in small samples based on CD11b expression. This served to avoid contamination by GBM and other cells prior to processing for RNA extraction and microarray analysis (Figure **1b**). Using a human genome survey microarray platform, we employed 32,878 probes for the interrogation of 29,362 human genes, of which 14,414 genes could be analyzed because they passed all quality control criteria (Figure **2**). Among these genes, 846 were affected by LPS stimulation, while the vast majority (13,548) were unaffected by LPS stimulation. Among these subsets of genes, we then identified genes that were differentially affected by co-culturing in the presence of a GBM tumor cell line, relative to stimulation by LPS alone. Among genes up-regulated by LPS stimulation, the majority were down-regulated in co-culture with GBM tumor cells. In contrast, co-culture with GBM tumor cells predominantly up-regulated genes among the subset that was unaffected by LPS stimulation. Finally, among genes affected by co-culture with the GBM tumor cell line and affected (136 genes) or unaffected by LPS stimulation (164 genes), we further identified those genes that were unaffected by co-culture with non-transformed, primary human astrocytes, resulting in two final subsets of 33 genes and 26 genes.

Review of the literature suggested an interesting candidate gene from each of the two final gene subsets based on their suggested roles in regulation of inflammation. Up-regulation of both AXL [18] and Caveolin-1 (CAV1) [19] has been demonstrated to reduce inflammation via inhibition of TNF-alpha production and were therefore investigated further. We confirmed up-regulation of these two molecules in an independent data set using freshly isolated monocytes from healthy donors (n=5). There was significant up-regulation of CAV1 and AXL when monocytes were stimulated with LPS in the presence of GBMs compared to stimulated monocytes alone as measured by quantitative PCR. The expression changes of CAV1 and AXL in monocytes in the presence of GBM were 43.6 and 5.3 fold (P=0.02, 0.04), respectively, compared to stimulated monocytes in the absence of GBM tumor cells. 

### Ex vivo analysis of tumor associated myeloid cells

**Figure 3:**
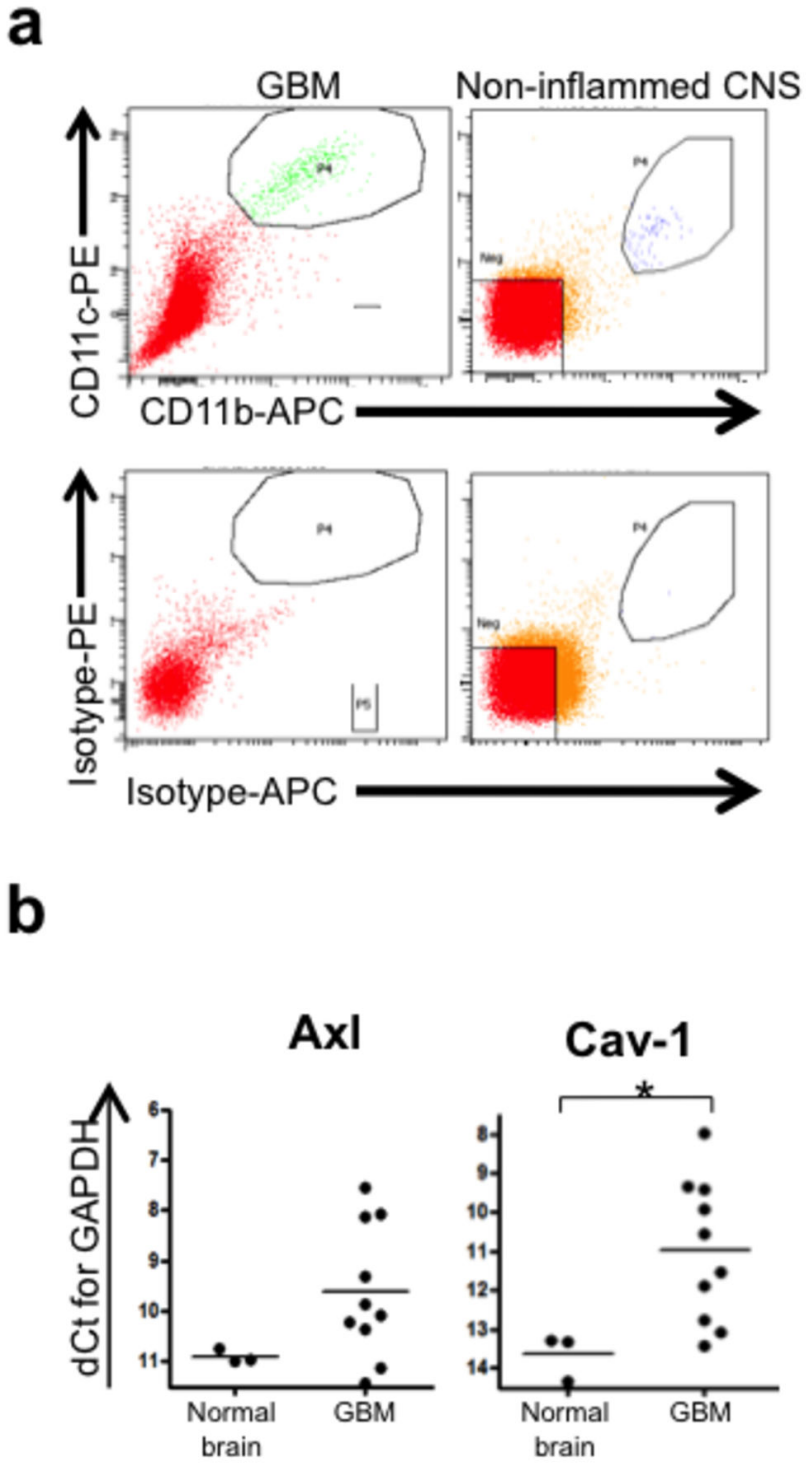
*Ex* *vivo* isolation and expression of AXL and CAV1 among myeloid cells in non-inflamed human CNS tissue and GBMs. (**a**) GBM tumor specimens or non-inflamed CNS tissue isolated from the anterior temporal tip during epileptic surgery were processed to single cell suspensions and stained with antibodies against CD11b and CD11c to sort infiltrating monocytes/microglia. (**b**) Quantitative PCR analysis of expression levels of AXL and CAV1 were determined for monocytes/microglia from *ex* *vivo* non-inflamed human CNS tissue (n=3) and primary GBMs (n=10).

Multiple lines of evidence both *in vitro* and in murine models point to the local tumor microenvironment as the major site where most immune modulation involving the myeloid compartment stem from [20]. However, comparable data in GBM patients are not well known. We therefore examined TAMs directly *ex vivo* from patients harboring primary GBMs (n=10) and normal brain (n=3). Immediately after surgical resection, fresh specimens were manually minced and reduced to single cell suspensions as previously described [12,17]. TAMs were isolated using FACS (CD11b+/CD11c+) (Figure **3a**), followed by RNA extraction, cDNA preparation and rtPCR amplification. Gene expression analysis demonstrated a statistically significant up-regulation of CAV1 in TAMs from patients with GBMs (6.32 fold) compared to normal brain (p= 0.03). AXL expression was also up-regulated *ex vivo* in TAMs from patients with GBMs (2.46 fold) compared to normal brain, but did not reach statistical significance (p = 0.1) (Figure **3b**). 

### Inhibition of CAV1 restores myeloid cell function as measured by TNF-α secretion

**Figure 4:**
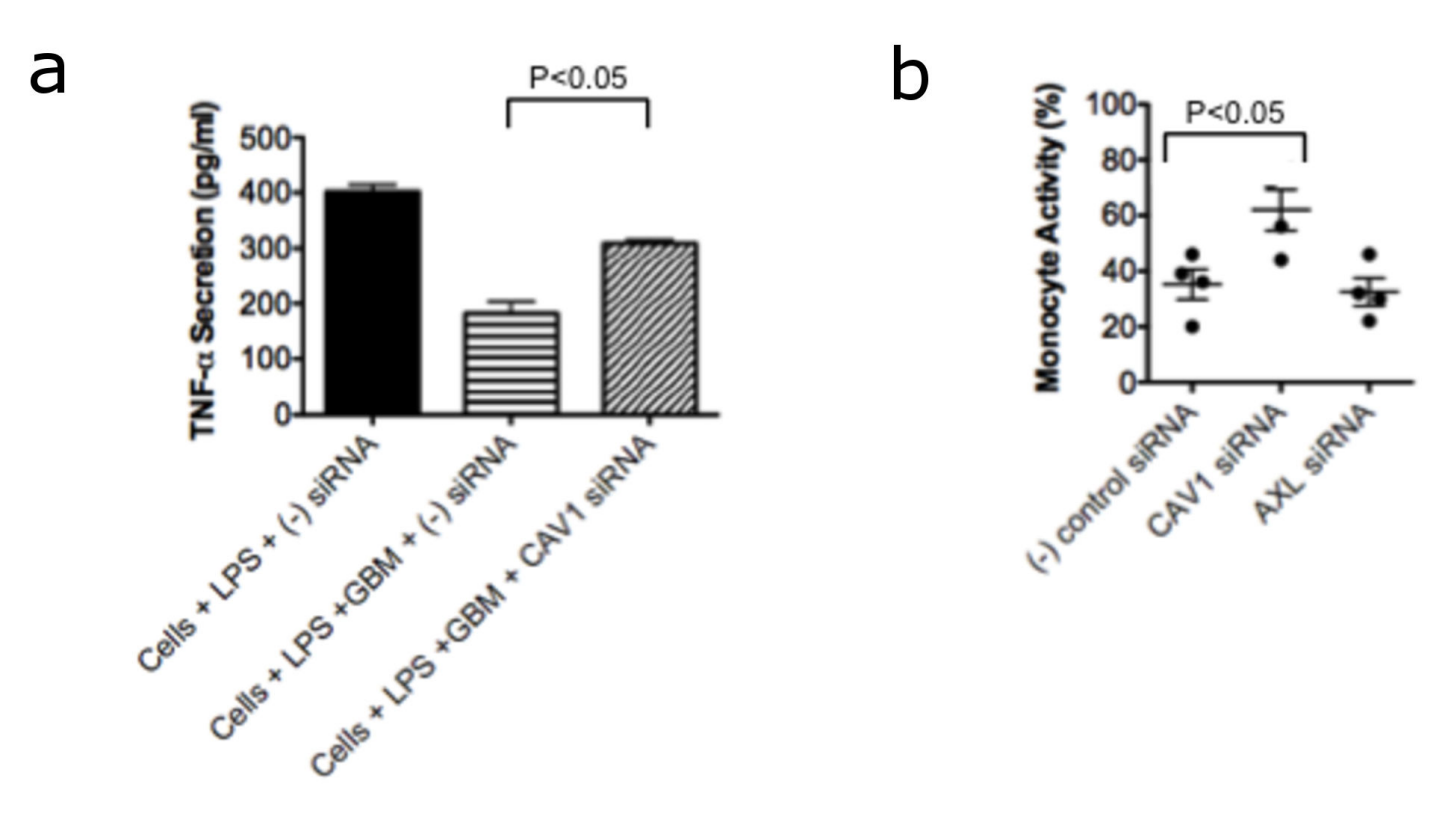
Restoration of myeloid cell function with siRNA inhibition of CAV1 but not AXL. (**a**) A representative example of the ability of GBM tumor cells to inhibit monocyte function, exemplified by TNF-alpha secretion after LPS stimulation, and the ability of CAV1 siRNA to reverse this inhibition. (**b**) Results from four independent experiments examining the ability of CAV1 and AXL siRNA inhibition to reverse GBM-mediated inhibition of TNF-alpha secretion. CAV1 suppression by siRNA significantly reverses GBM-mediated suppression and restores monocyte activity as measured by TNF-alpha secretion (p<0.05).

Our *ex vivo* analysis of TAMs validated up-regulation of CAV1 and to a lesser extent AXL in the local tumor microenvironment, but the functional significance of these findings was unclear. To determine whether these genes contributed to the GBM-mediated suppression of myeloid cells, we used siRNA inhibition to block CAV1 and AXL expression in our co-culture model. PMA stimulated monocytes were first transfected with siRNA specific for CAV1 or AXL, nonspecific siRNA, or left untransfected. siRNA transfection consistently reduced CAV1 and AXL gene expression by between 60-75% (data not shown). After 24 hours of co-culture in the presence or absence of GBM cells, supernatants were collected and TNF-alpha secretion was measured by ELISA as previously described [11,14]. Consistent with our previous data, there was a significant reduction in TNF-alpha secretion in LPS stimulated monocytes cultured in the presence of GBM tumor cells and transfected with nonsense siRNA (161pg/ml) compared to LPS stimulated monocytes in the absence of GBM tumor cells (414 pg/ml) (Figure **4a**). When siRNA specific for CAV1 was transfected in co-cultures of moncytes stimulated with LPS in the presence of GBM tumor cells, however, TNF alpha secretion increased by approximately 50% (315 pg/ml vs 161 pg/ml). Results obtained in four independent experiments demonstrated that transfection of monocytes with anti-CAV1 siRNA restored 42-78% of monocyte activity as measured by TNF-alpha secretion compared to transfection with non-specific siRNA (p=0.0278)(Figure **4b**). Importantly, transfection with siRNA specific for AXL did not reduce the GBM-mediated immunosuppression and restore TNF-alpha secretion, further supporting the specificity of CAV1 up-regulation to the observed myeloid cell suppression conferred by GBM tumor cells. 

CAV1 was initially described as a member of a family of scaffolding proteins that interacts with signaling molecules and regulates their activity [19]. It has been reported to have many functions, including the formation of caveolae, membrane trafficking, signal transduction pathways, apoptosis, calcium and lipid homeostasis in fibroblasts, adipocytes, and endothelial cells [21]. More recent evidence indicates that CAV1 also suppresses inflammation [22]. CAV1 expression has been demonstrated in multiple immune cells including monocytes/macrophages, dendritic cells, and lymphocytes [23,24]. Similar to our findings in human GBMs, upregulation of CAV1 in murine macrophages dramatically reduced pro-inflammatory cytokine production (TNF-alpha and IL-6) and increased anti-inflammatory cytokine production (IL-10) [19]. Reported mechanisms of CAV1 mediated immunosuppression in murine models include inhibition of eNOS activity [25] and activation of the MKK3/p38 pathway [19]. 

Taken together, these data indicate that GBM-mediated suppression of tumor-associated myeloid cell function is mediated at least in part by CAV1, and importantly, that activity can be restored by suppressing CAV1. Currently FDA approved pharmacological inhibitors of CAV1 such as lovastatin and celecoxib [26] may be useful in altering the local tumor microenvironment and augmenting current immunotherapy against human glioblastoma and a variety of other solid human tumors characterized by the presence of large numbers of TAMs.

## References

[1] Brooks WH, Markesbery WR, Gupta GD, Roszman TL (1978) Relationship of lymphocyte invasion and survival of brain tumor patients. Ann Neurol 4: 219-224. doi:10.1002/ana.410040305. PubMed: 718133. 718133

[2] Kuppner MC, Hamou MF, de Tribolet N (1988) Immunohistological and functional analyses of lymphoid infiltrates in human glioblastomas. Cancer Res 48: 6926-6932. PubMed: 3052809. 3052809

[3] Paine JT, Handa H, Yamasaki T, Yamashita J, Miyatake S (1986) Immunohistochemical analysis of infiltrating lymphocytes in central nervous system tumors. Neurosurgery 18: 766-772. doi:10.1227/00006123-198606000-00015. PubMed: 3488516. 3488516

[4] Ridley A, Cavanagh JB (1971) Lymphocytic infiltration in gliomas: evidence of possible host resistance. Brain 94: 117-124. doi:10.1093/brain/94.1.117. PubMed: 5552158. 5552158

[5] Saito T, Tanaka R, Yoshida S, Washiyama K, Kumanishi T (1988) Immunohistochemical analysis of tumor-infiltrating lymphocytes and major histocompatibility antigens in human gliomas and metastatic brain tumors. Surg Neurol 29: 435-442. doi:10.1016/0090-3019(88)90137-1. PubMed: 3259730. 3259730

[6] Yamanaka R (2008) Cell- and peptide-based immunotherapeutic approaches for glioma. Trends Mol Med 14: 228-235. doi:10.1016/j.molmed.2008.03.003. PubMed: 18403264. 18403264

[7] Filipazzi P, Huber V, Rivoltini L (2012) Phenotype, function and clinical implications of myeloid-derived suppressor cells in cancer patients. Cancer Immunol Immunother 61: 255-263. doi:10.1007/s00262-011-1161-9. PubMed: 22120756. 22120756 PMC11029611

[8] Hussain SF, Yang D, Suki D, Aldape K, Grimm E et al. (2006) The role of human glioma-infiltrating microglia/macrophages in mediating antitumor immune responses. Neuro Oncol 8: 261-279. doi:10.1215/15228517-2006-008. PubMed: 16775224. 16775224 PMC1871955

[9] Hussain SF, Yang D, Suki D, Grimm E, Heimberger AB (2006) Innate immune functions of microglia isolated from human glioma patients. J Transl Med 4: 15. doi:10.1186/1479-5876-4-15. PubMed: 16573834. 16573834 PMC1501057

[10] Hussain SF, Heimberger AB (2005) Immunotherapy for human glioma: innovative approaches and recent results. Expert Rev Anticancer Ther 5: 777-790. doi:10.1586/14737140.5.5.777. PubMed: 16221048. 16221048

[11] Kennedy BC, Maier LM, D'Amico R, Mandigo CE, Fontana EJ et al. (2009) Dynamics of central and peripheral immunomodulation in a murine glioma model. BMC Immunol 10: 11. doi:10.1186/1471-2172-10-11. PubMed: 19226468. 19226468 PMC2654428

[12] Anderson RC, Anderson DE, Elder JB, Brown MD, Mandigo CE et al. (2007) Lack of B7 expression, not human leukocyte antigen expression, facilitates immune evasion by human malignant gliomas. Neurosurgery 60: 1129-1136; discussion: 17538388. 17538388 10.1227/01.NEU.0000255460.91892.44

[13] Parney IF, Waldron JS, Parsa AT (2009) Flow cytometry and in vitro analysis of human glioma-associated macrophages. Laboratory investigation. J Neurosurg 110: 572-582. doi:10.3171/2008.7.JNS08475. PubMed: 19199469. 19199469 PMC3064468

[14] Kostianovsky AM, Maier LM, Anderson RC, Bruce JN, Anderson DE (2008) Astrocytic regulation of human monocytic/microglial activation. J Immunol 181: 5425-5432. PubMed: 18832699. 18832699 10.4049/jimmunol.181.8.5425

[15] Assanah M, Lochhead R, Ogden A, Bruce J, Goldman J et al. (2006) Glial progenitors in adult white matter are driven to form malignant gliomas by platelet-derived growth factor-expressing retroviruses. J Neurosci 26: 6781-6790. doi:10.1523/JNEUROSCI.0514-06.2006. PubMed: 16793885. 16793885 PMC6673823

[16] Held-Feindt J, Hattermann K, Müerköster SS, Wedderkopp H, Knerlich-Lukoschus F et al. (2010) CX3CR1 promotes recruitment of human glioma-infiltrating microglia/macrophages (GIMs). Exp Cell Res 316: 1553-1566. doi:10.1016/j.yexcr.2010.02.018. PubMed: 20184883. 20184883

[17] Anderson RC, Elder JB, Brown MD, Mandigo CE, Parsa AT et al. (2002) Changes in the immunologic phenotype of human malignant glioma cells after passaging in vitro. Clin Immunol 102: 84-95. doi:10.1006/clim.2001.5152. PubMed: 11781071. 11781071

[18] Sharif MN, Sosic D, Rothlin CV, Kelly E, Lemke G et al. (2006) Twist mediates suppression of inflammation by type I IFNs and Axl. J Exp Med 203: 1891-1901. doi:10.1084/jem.20051725. PubMed: 16831897. 16831897 PMC2118370

[19] Wang XM, Kim HP, Song R, Choi AM (2006) Caveolin-1 confers antiinflammatory effects in murine macrophages via the MKK3/p38 MAPK pathway. Am J Respir Cell Mol Biol 34: 434-442. doi:10.1165/rcmb.2005-0376OC. PubMed: 16357362. 16357362 PMC2644205

[20] Filipazzi P, Bürdek M, Villa A, Rivoltini L, Huber V (2012) Recent advances on the role of tumor exosomes in immunosuppression and disease progression. Semin Cancer Biol 22: 342-349. doi:10.1016/j.semcancer.2012.02.005. PubMed: 22369922. 22369922

[21] Anderson RG (1993) Caveolae: where incoming and outgoing messengers meet. Proc Natl Acad Sci U S A 90: 10909-10913. doi:10.1073/pnas.90.23.10909. PubMed: 8248193. 8248193 PMC47891

[22] de Almeida CJ, Witkiewicz AK, Jasmin JF, Tanowitz HB, Sotgia F et al. (2011) Caveolin-2-deficient mice show increased sensitivity to endotoxemia. Cell Cycle 10: 2151-2161. doi:10.4161/cc.10.13.16234. PubMed: 21670588. 21670588 PMC3154364

[23] Harris J, Werling D, Hope JC, Taylor G, Howard CJ (2002) Caveolae and caveolin in immune cells: distribution and functions. Trends Immunol 23: 158-164. doi:10.1016/S1471-4906(01)02161-5. PubMed: 11864845. 11864845

[24] Harris J, Werling D, Koss M, Monaghan P, Taylor G et al. (2002) Expression of caveolin by bovine lymphocytes and antigen-presenting cells. Immunology 105: 190-195. doi:10.1046/j.1365-2567.2002.01362.x. PubMed: 11872094. 11872094 PMC1782652

[25] Santizo RA, Xu HL, Galea E, Muyskens S, Baughman VL et al. (2002) Combined endothelial nitric oxide synthase upregulation and caveolin-1 downregulation decrease leukocyte adhesion in pial venules of ovariectomized female rats. Stroke 33: 613-616. doi:10.1161/hs0202.102363. PubMed: 11823678. 11823678

[26] Guruswamy S, Rao CV (2009) Synergistic effects of lovastatin and celecoxib on caveolin-1 and its down-stream signaling molecules: Implications for colon cancer prevention. Int J Oncol 35: 1037-1043. PubMed: 19787257. 19787257 10.3892/ijo_00000418

